# Validation of Analytical Results for Counter-Current Flow in Square Channels Separated by a Membrane in a Hemodialysis Module Using Experimental Module Results

**DOI:** 10.3390/membranes16050160

**Published:** 2026-04-30

**Authors:** Akram Abdullah, Rathinam Panneer Selvam

**Affiliations:** Department of Civil Engineering, University of Arkansas, Fayetteville, AR 72701, USA; rps@uark.edu

**Keywords:** counter-current flow, membrane, hemodialysis, 1D analytical solution, experimental results

## Abstract

Counter-current flow in channels separated by a membrane has been studied by several scientists and researchers. The current study aims to analytically simulate and describe the distribution of pressure, volumetric flow rate, and velocity in square channels separated by a membrane. Consequently, the study was conducted using one-dimensional (1D) analytical solutions to achieve several objectives: avoiding the execution of experimental tests, reducing the effort required for expensive and time-consuming module design, and enabling easy observation of variations in pressure, volumetric flow rate, and velocity. The 1D analytical solution directly simulates flow in square channels separated by a membrane by solving the continuity equation and Darcy’s law, through which pressure, volumetric flow rate, and velocity are calculated. Experimental results were used to validate the 1D analytical solutions. The results of the current study indicate that pressure decreases from the inlet to the outlet of the channel, while the horizontal velocity decreases from the inlet to the midpoint of the channel length and then increases toward the outlet. The 1D analytical solutions show acceptable accuracy when compared with experimental results. Consequently, these solutions can be used to explore and illustrate the distributions of pressure, volumetric flow rate, and velocity in square channels separated by a membrane, enabling the evaluation of hemodialysis prototype module performance and efficiency prior to fabrication.

## 1. Introduction

### 1.1. Background

Analytical solutions for flow between two porous parallel plates have been reported by several researchers. The Navier–Stokes (N–S) equations have been solved to obtain a complete description of one-dimensional, incompressible, steady-state laminar flow in a channel with a rectangular cross-section. One side of the cross-section, representing the distance between the porous walls, is much smaller than the other, with both walls being equally porous. This configuration leads to detailed expressions describing the dependence of velocity components and pressure [1]. An analytical solution for the pressure drop in a rectangular slit with constant wall velocity was reported, where the velocity is proportional to the transmembrane pressure difference (constant wall permeability), as well as for tubes with porous (permeable) walls under constant wall permeability conditions. The pressure drop in crossflow membrane modules is a function of wall permeability, channel dimensions, axial position, and fluid properties [2]. An analytical solution for one-dimensional incompressible fluid flow between two porous parallel plates was reported. Due to the porosity of the walls, a crossflow (vertical velocity) develops along the y-direction. The analytical solution was obtained by applying the continuity and Navier–Stokes (N-S) equations under specific assumptions and boundary conditions. The solution depends on the cross-stream Reynolds number [3]. Analytical solutions for two fluids flowing in parallel or countercurrent configurations through passages separated by a permeable wall using a one-dimensional (1D) model were derived. The 1D analytical model applies the continuity equation to describe flow within the channels and Darcy’s law to represent flow through the membrane, enabling the determination of mass transfer under countercurrent conditions. Countercurrent flow improves the uniformity of mass flux distribution. Under these conditions, the flow rate decreases from the inlet to an intermediate axial location and then increases again toward the outlet [4].

In membrane filtration processes where both free flow and porous flow occur, the Navier–Stokes (N-S) equations are used to model free flows, while Darcy’s law is applied to flows in porous media with low porosity. When free flow and porous flow coexist, continuity of the flow field across the interface between the laminar flow region and the porous region can be modeled by coupling Darcy’s law with the N-S equations. Recent studies have modeled fluid flow in hollow fiber membrane modules. Hemodialyzers are cylindrical modules filled with several thousand (~8000–16,000) hollow polymeric fibers and are commonly used in hemodialysis therapy devices. Typical hemodialyzer module dimensions range from 1 to 4 cm in diameter, membrane (fiber) thickness from 9 to 90 μm, membrane (fiber) diameter from 200 to 750 μm, and module length from 15 to 35 cm [4,5,6,7,8,9,10,11,12,13,14,15,16]. Square micro- and meso-scale channels are increasingly used in fabricated devices, but their corner-induced low-velocity and low-shear regions create flow and transport behavior fundamentally different from circular tubes or rectangular slits. These persistent corner effects invalidate tube- or slit-based models, requiring dedicated modeling approaches to accurately capture velocity distributions, solute transport, and scaling behavior in square geometries.

### 1.2. Performance, Efficiency, Removal, and Clearance of Hemodialysis

Membrane processes are used in technical and medical applications to separate waste from valuable substances [17]. Hollow fiber membrane modules are manufactured based on the fiber material used and the type of impurities they are designed to separate [18]. The effects of all dialyzer-related factors on solute clearance have not been fully reported in the literature, making it difficult to optimize dialyzer design to maximize solute removal from patients’ blood [13,19]. A crossflow process in which fluid is removed from blood by applying a suitable transmembrane pressure (convective mass transfer) and an appropriate concentration gradient (diffusive mass transfer) is known as hemofiltration (HF) and hemodialysis (artificial kidney) [17]. The efficiency and performance of hemodialysis operations depend on both module design and clinical procedures [10]. The typical operating conditions are achieved when the inlet blood and dialysate flow rates are 300 mL/min and 500 mL/min, respectively [14,15,20]. The removal efficiency in a single pass for low-molecular-weight metabolites such as urea is therefore approximately 65%, assuming that convection is the primary mass-transfer mechanism [5]. Membrane separation offers advantages such as low energy consumption, simplicity, high efficiency, and ease of operation and control [21]. The ideal dialysis membranes should achieve the following optimum characteristics as reported [21,22,23]:

1. Sufficient mechanical, physical (withstand pressure), chemical, and thermal stability (withstand the sterilization process).
2. Large surface area.
3. Optimal biocompatibility (hydrophilic/hydrophobic).
4. A highly porous support layer combined with a thin active separation layer (high solute flux) to ensure high permeability for efficient removal of toxins from the blood.
5. High macro-porosity (high hydraulic permeability).
6. Narrow and uniform pore-size distribution with a sharp molecular weight cut-off (MWCO) and high selectivity to prevent the loss of beneficial proteins.
7. No back diffusion (from the dialysate to the blood).
8. Minimal surface roughness to reduce interactions with blood.
9. Excellent hemocompatibility and cytocompatibility to prevent protein adsorption, platelet adhesion, blood coagulation, complement activation, and hemolysis.
10. Prevention of the entry of bacterial contaminants, such as endotoxins, from the dialysis fluid into the bloodstream to avoid adverse effects on patient health.

### 1.3. Objectives

The primary objectives are to physically describe the flow patterns that develop under counter-current conditions and to demonstrate the advantages of one-dimensional (1D) analytical solutions for the design and optimization of membrane contactors, ultimately saving time and cost compared with expensive experimental prototyping.

The main objective of the current study is to analytically simulate and describe the distributions of pressure, volumetric flow rate, and velocity in square channels separated by a membrane. The 1D analytical solutions are characterized by the following advantages:

1. The 1D analytical solutions can model and visualize flow conditions with acceptable accuracy compared to measurements obtained using physical equipment, while avoiding the execution of experimental tests, which face significant challenges in tiny channels and membranes.
2. The 1D analytical solutions reduce the cost and time associated with physical modeling and experimental testing and allow potential issues, such as backflow, to be identified before the fabrication of expensive physical modules.
3. The 1D analytical solutions make it easy to observe variations in pressure and horizontal velocity within the model, whereas such observations are very difficult to achieve in real experimental setups.

## 2. Materials and Methods

### 2.1. Hoskins et al. (2025) [24] Study

**Figure 1:**
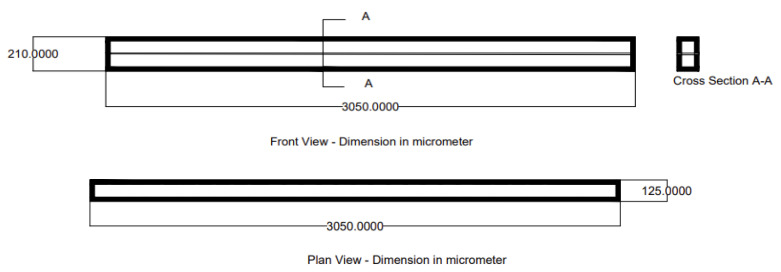
The experimental module [24] (front and plan view).

Membrane-integrated microfluidic chips with dimensions of 75 μm × 75 μm × 10 μm were fabricated. Burst-pressure testing was performed to evaluate the structural robustness of the fabricated devices. A steady input flow rate, supplied by a syringe pump using dyed water at 100 μL/min, was applied while three of the four inlet/outlet ports were sealed, resulting in an exponential increase in pressure over time. The results showed that the chips withstood internal pressures averaging 1.27 MPa. Flow testing over a range of approximately 35 μL/min to 345 μL/min confirmed stable operation in 75 μm square channels, with no leakage and minimal flow resistance up to approximately 175 μL/min, without deviation from the predicted behavior for the 75 μm channels. The fabricated membrane was experimentally examined for pressure robustness under different volumetric flow rates using a model with overall dimensions of 3050 × 210 μm, consisting of two channels (75 μm each) separated by a 10 μm membrane and surrounded by a 25 μm solid wall [24], as shown in Figure 1.

### 2.2. Hemodialysis Module

The current study reviews the limitations of the hemodialysis process and suggests criteria for selecting an optimal module that achieves high performance, efficiently removes pollutants from the blood, attains a high clearance percentage, and reduces the time required to complete the process. Clearance is defined as the volume of blood that must be completely purified per unit time to achieve a given solute removal rate [14].

***CL = (Q_Bi_ C_Bi_ − Q_Bo_ C_Bo_)/C_Bi_*** (1)

where

- Q_Bi_ and Q_Bo_ are the blood flow rates at the hemodialyzer inlet and outlet, respectively.
- C_Bi_ and C_Bo_ are the inlet and outlet solute concentrations in the blood, respectively.

The module [24], with its current dimensions, is not suitable for use in hemodialysis unless it is modified and fabricated into the proposed module of the present study, with a length of 25 cm and 300 channels, compactly arranged within a hemodialyzer. The proposed configuration consists of rectangular modules arranged as 25 modules along the x-axis and 12 modules along the y-axis, yielding overall dimensions of (25 × 0.0125 cm) × (12 × 0.0210 cm) = 0.3125 cm × 0.252 cm, corresponding to a total cross-sectional area of 0.07875 cm^2^. With appropriate inlet and outlet ports for blood and dialysate, this configuration may improve hemodialysis process efficiency (clearance) and reduce the time required for urea removal from patient blood.

**Figure 2:**
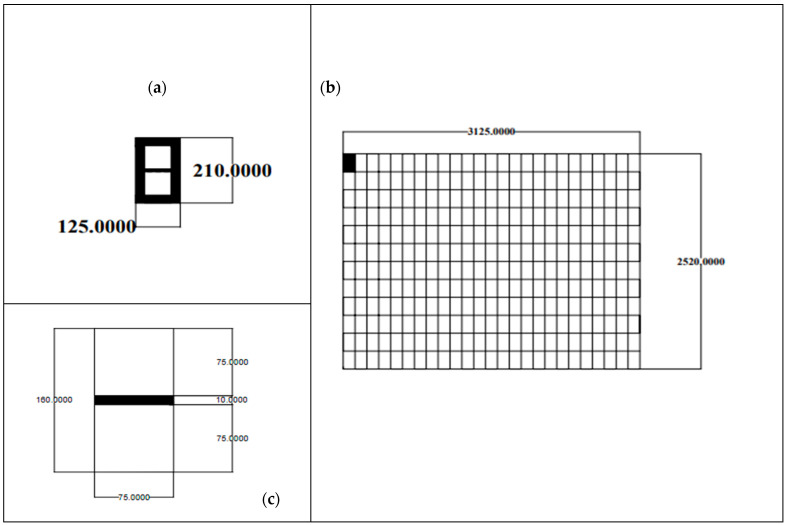
(**a**) 125 µm × 210 µm cross section of Hoskins module (**b**) Proposed 3125 µm × 2520 µm cross section (**c**) 75 µm × 160 µm cross section of Hoskins channels and membrane.

The proposed module is estimated to require 309 min to process 3000 mL of blood and 767 min for 7500 mL, with an average treatment time of approximately 538 min (9 h) at a flow velocity of *u* = 0.097 m/s within the module. A comparison between recent studies, the experimental study [24], and the proposed module of the current study (hemodialysis module) is presented in Table 1 and Figure 2.

## 3. Results

The experimental results for counter-current flow in square channels separated by a membrane were reported [24]. These results are tabulated and illustrated in the following sections.

### 3.1. Pressure Results

**Figure 3:**
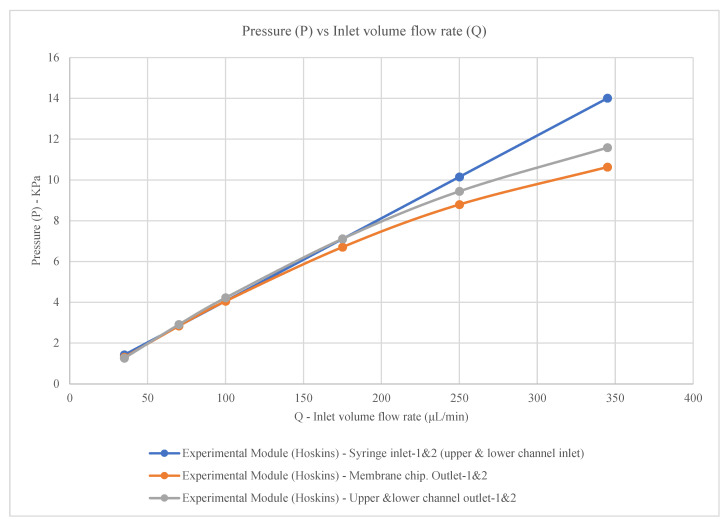
Pressure in experimental module (Hoskins) with constant wall permeability (model parameters are given in the text).

The experimental pressure was measured at syringe inlet 1 and inlet 2 (upper and lower channel inlets), membrane chip outlet 1 and outlet 2, and upper and lower channel outlet 1 and outlet 2 by Hoskins et al. The measured pressures follow the empirical equations presented in Table 2 and Figure 3.

The pressure for the syringe inlets 1 and 2 (upper and lower channel inlets)

**P = 0.0406Q** (2)

The pressure for the membrane chip outlets 1 and 2

**P = −0.00005Q^2^ + 0.0491Q − 0.3561** (3)

The pressure for the upper and lower channel outlets 1 and 2

**P = −0.00005Q^2^ + 0.0523Q − 0.5086** (4)

where

P = pressure (kpa);
Q = inlet volume flow rate (μL/min).

### 3.2. Velocity Results

**Figure 4:**
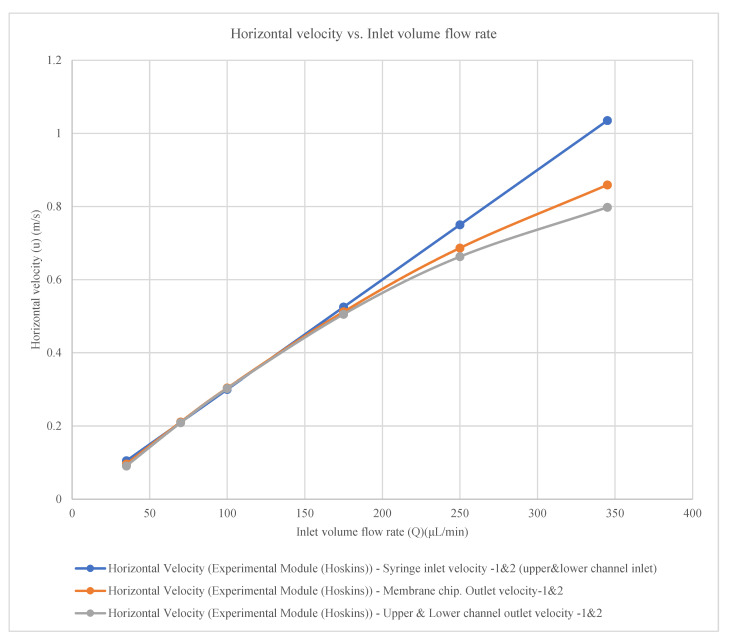
Horizontal velocity in experimental module [24] with constant wall permeability (model parameters are given in the text).

The experimental horizontal velocity was measured at syringe inlet 1 and inlet 2 (upper and lower channel inlets), membrane chip outlet 1 and outlet 2, and upper and lower channel outlet 1 and outlet 2 [24]. The measured velocities follow the empirical equations presented in Table 3 and Figure 4.

The horizontal velocity for the syringe inlets 1 and 2 (upper and lower channel inlets)

**u(m/s) = 0.003Q + 4 × 10^−16^** (5)

The horizontal velocity for the membrane chips. outlets 1 and 2

**u(m/s) = −3 × 10^−6^Q^2^ + 0.0036Q − 0.026** (6)

The horizontal velocity for the upper and lower channels outlets 1 and 2

**u(m/s) = −4 × 10^−6^Q^2^ + 0.0038Q − 0.0372** (7)

where u = horizontal velocity (m/s);

Q = inlet volume flow rate (μL/min).

## 4. Comparison of Results from Hagen–Poiseuille, Babu V. (2022) [3], Hoskins et al. (2025) [24] Experiment, and the 1D Analytical Solution

### 4.1. Calculation of Permeability (k), Porosity, and Hydraulic Permeability (Lp) for Square Channels in Hoskins et al. (2025) [24] Study

#### 4.1.1. Calculation of the Permeability (k)

The Hagen–Poiseuille (HP) equation for a square cross-section channel was used [24].

∆P = 0.63 × 8 *µ*QL/π (a/2)^4^

where a is the height and width of the square channel (m).

The mean velocity in the channel can be calculated from Hagen–Poiseuille (HP) equation as

**u_mean/HP_ = [(dp/dx) π (a^2^)]/80.64 *µ***

The mean velocity through the membrane can be calculated from Darcy’s law

**u_mean_
_/Darcy_ = (k/μ) ∂p/∂x**

By assuming that **u_mean/HP_ = u_mean__/Darcy_** we obtain

(**dp/dx**) **π** (**a^2^**)**/80.64 *μ* =** (**k/μ**) **∂p/∂x,** which yields

**k = π a^2^/80.64**

For a = h =7.5 × 10^−5^ m

k = π (7.5 × 10^−5^^2)/80.64 = **2.19 × 10^−10^ m^2^**

#### 4.1.2. Calculation of Porosity

Membranes with thickness of 10 µm and an area of 75 × 75 µm^2^ containing pores with a diameter of 3.73 µm and very high porosity reaching up to 60% were fabricated [24]. Each pore is modeled as a square opening with a side length equal to the pore width (3.73 µm) plus the wall thickness (1 µm), resulting in an effective pitch width w = 4.73 µm.

**Porosity** is defined as the ratio of the volume of voids to the total volume:

**(ɸ) = volume of voids/total volume**

The number of pores is calculated as Number of pores = (3000/4.73) × (75/4.73) = 10,057.

The volume of a single pore is:

volume of a one pore = 3.73 × 3.73 × 10= 139.129 µm^3^

**Therefore, the porosity is** (**ɸ**) = volume of voids/total volume = (10,057 × 139.129)/3000 × 75 × 10 = **0.622**

#### 4.1.3. Calculation of the Hydraulic Permeability (Lp)

The general equation that relates the change in the volumetric flow rate in the channel to the pressure and flux through the membrane is

**dQ/dx = w × q(x) = w × L_p_ × P_i_**

To calculate the hydraulic permeability (L_p_), parameters obtained from the experimental results are used [24].

Inlet volumetric flow rate (Q_i_) = 345 μL/min= 5.75 × 10^−9^ m^3^/s

Outlet volumetric flow rate (Q_o_) = 270 μL/min = 4.50 × 10^−9^ m^3^/s

Channel length (L) = 3 × 10^−3^ m

Channel width (w) = 7.5 × 10^−5^ m

Inlet pressure (P_i_) = 14.145 KPa

dQ/dx = w × q(x) = (Q_i_ − Q_o_)/L = w × q(x), Substituting the known values:

((5.75 × 10^−9^ − 4.50 × 10^−9^) μL/min)/3 × 10^−3^ = 7.5 × 10^−5^ × q(x), which yields: q(x) = 5.56 × 10^−3^ m/s

Finally, the hydraulic permeability is calculated as: Lp = q(x)/P_i_ = 5.56 × 10^−3^/14,145 = **3.93 × 10^−7^ m/Pa·s**

The permeability (k), porosity, and hydraulic permeability (Lp) parameters of the module [24] are presented in Table 4.

The model experimentally was examined by applying an inlet volumetric flow rate (Q) and measuring the outlet volumetric flow rate. From these measurements, they calculated the mean velocity (Q/A) and determined the pressure gradient along with the model length. To validate the one-dimensional (1D) analytical solutions, the current study used the experimental results [24].

**Figure 5:**
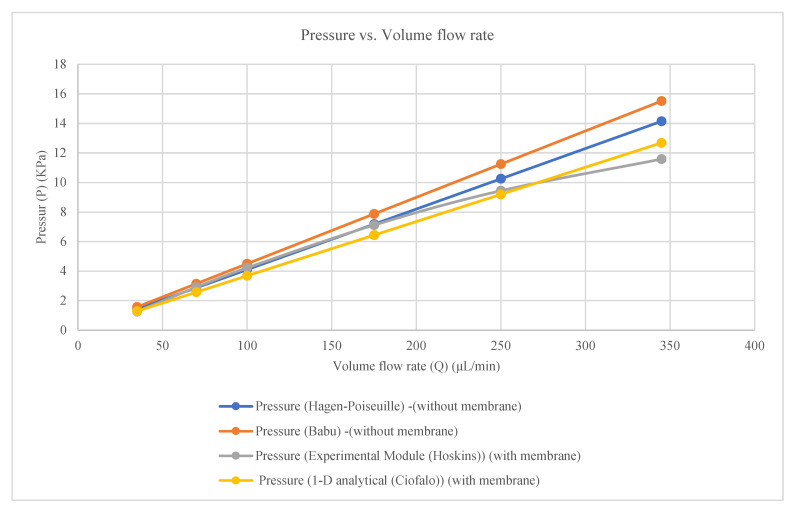
Comparison of pressure drop in a square channel between the Hagen–Poiseuille and Babu models (impermeable walls) and the Hoskins experimental results with the 1D analytical solution (Ciofalo) assuming constant wall permeability (model parameters are given in the text).

**Figure 6:**
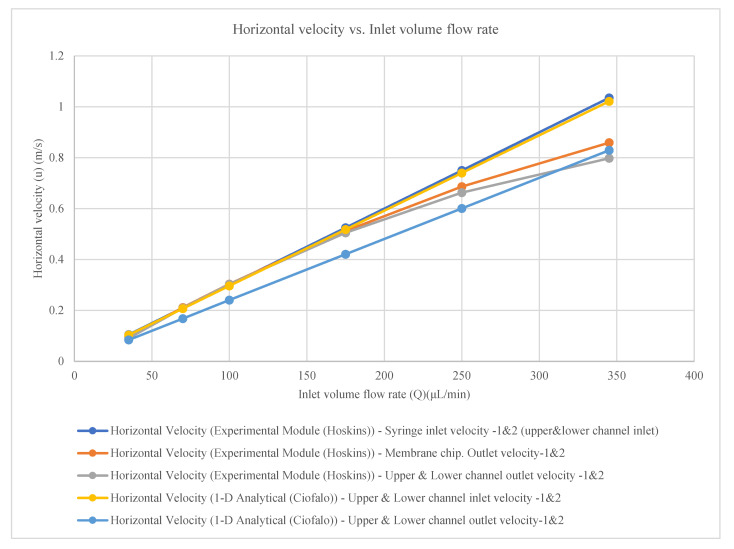
Comparison of the horizontal velocity drop in a square channel between the Hagen–Poiseuille model with impermeable walls, Hoskins’ experimental results, and the one-dimensional (1D) analytical solution (Ciofalo) with constant wall permeability (model parameters are provided in the text).

The theoretical results were calculated for a square solid channel without considering the membrane effect using the Hagen–Poiseuille equation, whereas the experimental results were obtained from a fabricated physical model of countercurrent flow in channels separated by a membrane [24]. For further comparison, theoretical results are also calculated for a solid square channel without membrane effects using the proposed expression [3]. One-dimensional (1D) analytical solutions for counter-current flow in channels separated by a membrane were developed based on specific assumptions [4]. The pressure (P), volumetric flow rate (Q) and horizontal velocity comparisons for the theoretical and experimental results [24] together with the 1D analytical (theoretical) results are presented in Table 5 and Table 6 and Figure 5 and Figure 6.

**P = (28.46μ Q_i_ L)/(h^4^)** (8)

where P = pressure (KPa);

Q_i_ = inlet volume flow rate (m^3^/s);

L = channel length (m) = 0.003 m;

h = channel height (m) = 75 × 10^−6^ m;

μ = dynamic viscosity (Pa.s) = 0.001 Pa.s.

The comparison between the Hagen–Poiseuille and Babu (theoretical) results, the Hoskins experimental results, and the 1D analytical (theoretical) results is shown in Table 5.

To provide an objective assessment of model accuracy, the agreement between analytical predictions and experimental measurements is quantified using the root-mean-square error (RMSE) for pressure, axial velocity, and volumetric flow rate. To facilitate comparison across operating conditions and variable magnitudes, a normalized RMSE (NRMSE) is additionally reported. Applied to the present data set, the resulting NRMSE values remain below 6% for pressure distributions, below 8% for axial-averaged velocity and below 3% for volumetric flow rate, indicating good predictive accuracy at the module scale.

## 5. Analysis and Discussion

The membrane-integrated chips exhibited outlet flow asymmetries greater than 10%, indicating active fluid transfer across the membrane and highlighting flow-dependent permeability [24]. The theoretical results were calculated for a square solid channel without considering the membrane effect using the Hagen–Poiseuille equation and the proposed expression. The results show slight differences because of the effect of horizontal velocity variations in the *y* and *z* directions (∂u/∂y and ∂u/∂z) for a rectangular cross-section channel [3]. The experimental results measured the flow parameters for counter-current flow in channels separated by a membrane [24]. A one-dimensional (1D) analytical solution for counter-current flow in channels separated by a membrane was derived based on specific assumptions [4]. The experimental results [24] and the 1D analytical solutions [4] show slight differences; however, these differences remain within an acceptable range.

The one-dimensional analytical model is valid for low-Reynolds-number, steady laminar flow in long, uniform channels with Newtonian fluids, but its accuracy decreases at higher flow rates, for non-Newtonian behavior, and under scale-up conditions. Thus, it should be viewed as a tool for capturing fundamental trends at the module scale rather than providing direct quantitative predictions for clinical-scale designs. Dyed water was used for flow visualization, providing Newtonian properties suitable for capturing qualitative laminar flow patterns, though its properties differ from non-Newtonian blood. Consequently, while observed transport trends remain representative, higher-pressure losses and altered shear stresses are expected with blood, necessitating future validation using blood-analog or blood-mimicking fluids for clinical relevance. Flow rates in the analytical model were matched to experimental conditions, and minor differences in volumetric flow rate do not affect the qualitative trends. However, at flow rates above ~175 μL/min, deviations increase as the assumptions of fully developed, laminar, one-dimensional flow break down due to entrance effects, increased shear, and emerging three-dimensional flow features.

A quantitative error analysis was examined to clarify model accuracy and operational bounds. Comparison with experiments using normalized RMSE shows errors below 6% for pressure, 8% for axial velocity, and 3% for volumetric flow rate within the validated regime, with deviations attributed to entrance effects and cross-sectional nonuniformities inherent to the 1D formulation. The model is now explicitly limited to laminar square-channel flow (Re ≲ 200) and pressures below membrane compaction, beyond which additional physics must be considered.

The results presented in Table 5 and Figure 5 and Figure 6 lead to the following remarks:

1. There is an approximate 10% difference between the theoretical results calculated using the Hagen–Poiseuille equation [24] and those obtained for a solid square channel [3]. This difference arises from the methods used to solve the second-order differential equations and the assumptions involved. A Hagen–Poiseuille equation modified from a circular to a square cross-section was employed [24]; a Fourier-series solution was used to solve the second-order differential equations for rectangular sections [3].
2. Differences are also observed between the experimental results measured [24] and the one-dimensional (1D) analytical solution proposed for flow in square channels separated by a membrane [4]. These differences can be attributed to the fact that precise experimental instruments were utilized to measure pressure and volumetric flow rate, despite the challenges associated with measurements in micrometer-scale channels [24], whereas analytical solutions that involve simplifying assumptions, which influence the results [4].
3. The experimental results for pressure, volumetric flow rate, and horizontal velocity reported agree well with the theoretical results up to a specific inlet volumetric flow rate of 175 μL/min. Beyond this value, the experimental results deviate and decrease with increasing flow rate [24], whereas the one-dimensional (1D) analytical solution continues to increase at a constant rate as the volumetric flow rate increases [4].

The current model therefore remains applicable as a hydrodynamic framework, with Newtonian validation establishing its fundamental accuracy. Non-Newtonian blood effects are expected to be incorporated through effective viscosity or shear-dependent extensions, while the inclusion of shear-rate-dependent viscosity and hematocrit effects is identified as an important direction for future clinical-scale predictions. The present 1D steady-state formulation further assumes constant membrane permeability to establish an idealized hydrodynamic baseline and does not account for time-dependent phenomena such as protein adsorption, concentration polarization, or membrane fouling, which are likewise identified as important directions for future work.

## 6. Conclusions

The results of the current study agree with the experimental results for counter-current flow in square channels separated by a membrane within acceptable accuracy [24]. Therefore, the experimental results reported [24] can be used to validate the one-dimensional (1D) analytical solution developed [4]. Consequently, the 1D analytical solutions for counter-current flow in square channels separated by a membrane can be employed to explore and visualize the distributions of pressure, volumetric flow rate, and velocity with acceptable accuracy [4]. This approach enables the evaluation of hemodialyzer prototype module performance and efficiency prior to fabrication, thereby reducing both time and cost by eliminating the need for extensive experimental testing. In addition, the current study developed an Excel spreadsheet to calculate pressure, horizontal velocity, and volumetric flow rate for arbitrary module dimensions and fluid properties, allowing rapid assessment of module performance before fabrication. While manifold-induced maldistribution may arise in large channel arrays, the 1D model remains valid at the channel scale provided that channel hydraulic resistance dominates over manifold pressure variations, with full 3D CFD optimization deferred to future work.

The model does not eliminate the need for experimental validation but instead minimizes repeated testing within a well-defined laminar operating regime. Within these bounds, the one-dimensional analytical solution provides quantitatively accurate predictions of pressure and flow distributions, enabling reliable engineering-level module design and parametric analysis, while higher-fidelity experiments or simulations remain essential when operating beyond the prescribed limits. Quantitative RMSE analysis confirms that, within the defined laminar operating envelope, the 1D model predicts pressure, velocity, and volumetric flow rate with errors below 10%, providing engineering-level accuracy while significantly reducing experimental burden. RMSE analysis of pressure, velocity, and volumetric flow rate distributions demonstrates that the 1D model predicts module-scale behavior with errors below 10% within its laminar operating envelope, supporting its use as a practical design and screening tool.

The current study proposes a hemodialyzer module in which the cross module [24] can be utilized with several modifications, including increasing the length from 3 mm to 25 cm, increasing the number of channels to 300 (upper channels for blood and lower channels for dialysate), and increasing the velocity in the lower (dialysate) channel to account for the different flow rates of blood (300 mL/min) and dialysate (500 mL/min). These modifications may improve the efficiency of the hemodialysis process and reduce the time required to remove urea from patient blood. The average total blood volume in humans ranges from approximately 3000 mL to 7500 mL.

The final dimensions and parameters of the proposed hemodialyzer module (shown in Table 1 and Figure 2) in the current study are as follows:

Length = 25 cm;
Height = 0.252 cm;
Width = 0.3125 cm;
Number of modules (based on the module [24]) = 300;
Blood velocity = 0.097 m/s;
Dialysate velocity = (5/3) × 0.097 m/s = 0.1617 m/s.

The limitations that should be addressed for the proposed module (hemodialyzers) are outlined as follows:

- The blood flow rate through the hemodialyzer is approximately 300 mL/min from the human body.
- The average total blood volume in humans ranges from 3000 mL to 7500 mL, requiring approximately 10–15 min for treatment during hemodialysis.
- The dialysate flow rate is 500 mL/min; therefore, the channel cross-sectional area should be larger than that of the blood channel by a factor of 5/3 or, alternatively, the dialysate velocity should be increased by the same factor.
- The membrane surface area should be maximized (in both length and width) to increase the contact area and residence time between blood and dialysate solutes.
- Typical hemodialyzer module dimensions range from 2 to 5 cm in diameter and from 15 to 35 cm in length.
- Hemodialysis is a microfiltration process that operates under a pressure difference of 1000 to 20,000 Pa, with membrane pore sizes ranging from 0.05 to 10 μm and membrane thicknesses between 10 and 150 μm. The pressure difference between the two channels drives mass transfer between blood and dialysate through the membrane.

The criteria required for proper dialysis are assessed through positive clinical outcomes and laboratory tests. In addition, ideal dialysis membranes should exhibit optimal characteristics that ensure effective removal of waste products and excess fluid while maintaining overall patient health. These criteria include well-controlled blood pressure, adequate urea clearance (at least 1.2), and a urea reduction ratio of approximately 65% for thrice-weekly hemodialysis. Hemodialysis performance depends on blood pressure and mass balance, where the volumetric flow rate of blood containing urea at the inlet equals the flow rate of cleaned blood at the outlet. Solute clearance is the primary performance parameter evaluated in hemodialysis.

## Figures and Tables

**Table 1:** Comparison of hemodialysis modules among recent studies, the experimental [24], and the current study.

Module Parameter	Recent Studies	Experimental Module [24]	Current Study Module
Length (cm)	15–35	0.3	25
Membrane thickness (μm)	9–90	10	10
Height (Diameter (D) or Side) (μm)	Circular, 200–230	Rectangular (width × height): 75 × (75 + 10 + 75)	Rectangular (width × height): 75 × (75 + 10 + 75)
Number of modules in cross-section	8000–16,000	1	300
Blood flow rate (Q) (mL/min)	300	35–345 (dyed water) (ρ = 998 kg·m^−3^)	300
Dialysis flow rate (Q) (mL/min)	500	35–345 (dyed water) (μ = 1.0 mPa·s)	500

**Table 2:** Pressure in experimental module [24] with constant wall permeability.

Inlet Volume Flow Rate (Q)	Pressure (P) (KPa)		
Q (μL/min)	Syringe Inlets 1 and 2 (Upper and Lower Channel Inlets)	Membrane Chip Outlets 1 and 2	Upper and Lower Channel Outlets 1 and 2
35	1.421	1.30115	1.26065
70	2.842	2.8359	2.9074
100	4.06	4.0539	4.2214
175	7.105	6.70515	7.11265
250	10.15	8.7939	9.4414
345	14.007	10.63215	11.58365

**Table 3:** Horizontal velocity in experimental module [24] with constant wall permeability.

	Horizontal Velocity (u) (m/s)		
Inlet Volume Flow Rate (Q)	Experimental Module (Hoskins)		
Q (μL/min)	Syringe Inlets 1 and 2 (Upper and Lower Channels Inlets)	Membrane Chips Outlets 1 and 2	Upper and Lower Channels Outlets 1 and 2
35	0.105	0.096325	0.0909
70	0.21	0.2113	0.2092
100	0.3	0.304	0.3028
175	0.525	0.512125	0.5053
250	0.75	0.6865	0.6628
345	1.035	0.858925	0.7977

**Table 4:** Permeability (k), porosity, and hydraulic permeability (Lp) parameters of the [24] module.

Parameters	Permeability (k) m^2^	Porosity (ɸ) -	Hydraulic Permeability (Lp) m/Pa·s
Experimental Module	2.19 × 10^−10^	0.622	3.93 × 10^−7^

**Table 5:** Comparison of Hoskins’ theoretical and experimental results with the one-dimensional (1D) analytical solution.

Methods	Analytical (Hagen–Poiseuille)	Analytical (Babu)	Experiment Module (Hoskins)		1D Analytical Solution (Ciofalo)	
Limitation	without membrane		with membrane			
Input Data	Theoretical Results	Theoretical Results	Experimental Results		Theoretical Results k = 2.19 × 10^−10^ m^2^, Lp = 3.93 × 10^−7^ m/Pa.s	
Q = volume flow rate	Calculated	Calculated	Measured	Measured	Calculated	Calculated
Q_i_ (μL/min)	P_i_ (KPa)	P_i_ (KPa)	Q_o_ (μL/min)	P_o_ (KPa)	Q_o_ (μL/min)	P_i_ (KPa)
35	1.435	1.5739	35	1.26065	28.41	1.287
70	2.87	3.1477	70	2.9074	56.82	2.574
100	4.1	4.4967	100	4.2214	81.33	3.684
175	7.175	7.8693	175	7.11265	142.04	6.434
250	10.25	11.2419	218	9.4414	202.91	9.192
345	14.145	15.5138	270	11.58365	280.02	12.684

**Table 6:** Comparison of horizontal velocity between Hoskins’ experimental results and the 1D analytical solution.

	Horizontal Velocity (u) (m/s)				
Inlet Volume Flow Rate (Q)	Experimental Module (Hoskins)			1D Analytical Solution (Ciofalo)	
Q_i_ (μL/min)	Syringe Inlets 1 and 2 (Upper and Lower Channels Inlets)	Membrane Chips. Outlets 1 and 2	Upper and Lower Channels Outlets 1 and 2	Upper and Lower Channels Inlets 1 and 2	Upper and Lower Channel Outlets 1 and 2
35	0.105	0.096325	0.0909	0.103612	0.084096
70	0.21	0.2113	0.2092	0.207224	0.168193
100	0.3	0.304	0.3028	0.296625	0.240756
175	0.525	0.512125	0.5053	0.518059	0.420482
250	0.75	0.6865	0.6628	0.740089	0.600693
345	1.035	0.858925	0.7977	1.021314	0.828949

## Data Availability

The data presented in this study is available on request from the corresponding author.

## Nomenclature

P	pressure (N/m^2^= kg/m·s^2^ = Pascal)
U, u	horizontal velocity (m/s)
L	channel length (m)
h = 2H	channel height (m)
w	channel width (m)
Q	flow rate (m^3^/s)
K	permeability (m^2^)
Lp	hydraulic permeability (m/Pa·s)
u, v, w	velocity components in x, y, z direction (m/s)
RMSE	root-mean-square error
NRMSE	normalized root-mean-square error
Greek letters	µm micrometer
ρ	Density (kg/m^3^)
μ	Dynamic Viscosity (kg/m·s) (mPa·s)
ν = μ/ρ	Kinematic Viscosity (m^2^/s)
ɸ	Porosity (-)

## References

[1] Berman A.S. (1953). Laminar Flow in Channels with Porous Walls. J. Appl. Phys..

[2] Karode S. (2001). Laminar Flow in Channels with Porous Walls, Revisited. J. Membr. Sci..

[3] Babu V. (2022). Fundamentals of Incompressible Fluid Flow.

[4] Ciofalo M. (2023). Flow Through Parallel Channels Separated by a Permeable Wall. Thermofluid Dynamics.

[5] Labicki M., Piret J.M., Bowen B.D. (1995). Two-Dimensional Analysis of Fluid Flow in Hollow-Fibre Modules. Chem. Eng. Sci..

[6] Legallais C., Catapano G., Von Harten B., Baurmeister U. (2000). A theoretical model to predict the in vitro performance of hemodiafilters. J. Membr. Sci..

[7] Liao Z., Klein E., Poh C.K., Huang Z., Lu J., Hardy P.A., Gao D. (2005). Measurement of hollow fiber membrane transport properties in hemodialyzers. J. Membr. Sci..

[8] Ding W., He L., Zhao G., Zhang H., Shu Z., Gao D. (2004). Double porous media model for mass transfer of hemodialyzers. Int. J. Heat Mass Transf..

[9] Ding W., Li W., Sun S., Zhou X., Hardy P.A., Ahmad S., Gao D. (2015). Three-dimensional simulation of mass transfer in artificial kidneys. Artif. Organs.

[10] Abaci H.E., Altinkaya S.A. (2010). Modeling of hemodialysis operation. Ann. Biomed. Eng..

[11] Lu J., Lu W.-Q. (2010). A numerical simulation for mass transfer through the porous membrane of parallel straight channels. Int. J. Heat Mass Transf..

[12] Kim J.C., Cruz D., Garzotto F., Kaushik M., Teixeria C., Baldwin M., Baldwin I., Nalesso F., Kim J.H., Kang E. (2013). Effects of dialysate flow configurations in continuous renal replacement therapy on solute removal: Computational modeling. Blood Purif..

[13] Donato D., Boschetti de Fierro A., Zweigart C., Kolb M., Eloot S., Storr M., Krause B., Leypoldt K., Segers P. (2017). Optimization of dialyzer design to maximize solute removal with a two-dimensional transport model. J. Membr. Sci..

[14] Cancilla N., Gurreri L., Marotta G., Ciofalo M., Cipollina A., Tamburini A., Micale G. (2022). A porous media CFD model for the simulation of hemodialysis in hollow fiber membrane modules. J. Membr. Sci..

[15] Cancilla N., Gurreri L., Marotta G., Ciofalo M., Cipollina A., Tamburini A., Micale G. (2022). Performance Comparison of Alternative Hollow-Fiber Modules for Hemodialysis by Means of a CFD-Based Model. Membranes.

[16] Elahi A., Chaudhuri S. (2023). Computational Fluid Dynamics Modeling of the Filtration of 2D Materials Using Hollow Fiber Membranes. ChemEngineering.

[17] Raff M. (2022). Mass Transfer Models in Membrane Processes Applications in Artificial Organs.

[18] Lim K., Wang P., An H., Yu S. (2017). Computational Studies for the Design Parameters of Hollow Fibre Membrane Modules. J. Membr. Sci..

[19] Ronco C., Clark W.R. (2018). Haemodialysis membranes. Nat. Rev. Nephrol..

[20] Cancilla N., Gurreri L., Marotta G., Ciofalo M., Cipollina A., Tamburini A., Micale G. (2021). CFD prediction of shell-side flow and mass transfer in regular fiber arrays. Int. J. Heat Mass Transf..

[21] Sheng D., Li X., Sun C., Zhou J., Feng X. (2023). The Separation Membranes in Artificial Organs. Mater. Chem. Front..

[22] Wilson I.D. (2002). Encyclopedia of Separation Science.

[23] Stamatialis D.F., Papenburg B.J., Gironés M., Saiful S., Bettahalli S.N.M., Schmitmeier S., Wessling M. (2008). Medical applications of membranes: Drug delivery, artificial organs and tissue engineering. J. Membr. Sci..

[24] Hoskins J.K., Pysz P.M., Stenken J.A., Zou M. (2025). Multiscale 2PP and LCD 3D Printing for High-Resolution Membrane-Integrated Microfluidic Chips. Nanomanufacturing.

